# Opening the black box of registration practice for self-harm and suicide attempts in emergency departments: a qualitative study

**DOI:** 10.1186/s12875-024-02393-6

**Published:** 2024-04-27

**Authors:** Sarah Grube Jakobsen, Pernille Tanggaard Andersen, Jens Lauritsen, Christina Petrea Larsen, Elsebeth Stenager, Erik Christiansen

**Affiliations:** 1 Department of Regional Health Research, University of Southern Denmark, Unit of Mental Health Services, Aabenraa, Denmark; 2 Centre for Suicide Research, Bangs Boder 28-30, st. th, Odense, 5000 Denmark; 3 Department of Public Health, Unit for Health Promotion Research, University of Southern Denmark, Esbjerg, Denmark; 4 Department of Clinical Medicine, Unit of Orthopaedic Surgery, University of Southern Denmark, Odense University Hospital, Odense, Denmark

**Keywords:** Self-injurious Behaviour, Suicide, attempted, Emergency Department, Qualitative research, Diagnosis, Practice guidelines as topic

## Abstract

**Background:**

The World Health Organization has called for improved surveillance of self-harm and suicide attempts worldwide to benefit suicide prevention programs. International comparisons of registrations are lacking, however, and there is a need for systematically collected, high-quality data across countries. The current study investigated healthcare professionals’ perceptions of registration practices and their suggestions for ensuring high-quality registration of self-harm and suicide attempts.

**Methods:**

Qualitative interviews (*N* = 20) were conducted among medical secretaries, medical doctors, nurses, and registration advisers from psychiatric and somatic emergency departments in all regions of Denmark between September 2022 and March 2023. Content analysis was performed using NVivo.

**Results:**

Despite great efforts to standardize and assure the quality of registration in Denmark, almost all the healthcare professionals perceived registration practice as inconsistent and unreliable. Codes are often misclassified or unused due to insufficient time, non-standardized training, or insufficient information. The interview informants suggested that coding guidelines should be simplified and made more visible, alongside technical solutions in the electronic health record system.

**Conclusion:**

The study findings resulted in eight overall recommendations for clinical practice that aim at improving the registration of patients presenting with self-harm or suicide attempts. This would be expected to help improve surveillance and prevention programs.

**Supplementary Information:**

The online version contains supplementary material available at 10.1186/s12875-024-02393-6.

## Background

Suicidal behaviour is a major concern in many countries. Over 700,000 people die by suicide globally every year [1], and while suicide attempts have an even higher incidence, the numbers are difficult to determine. The World Health Organization (WHO) has called for improved surveillance of self-harm and suicide attempts to benefit suicide prevention programs [1, 2]. Real-time monitoring of data is also important for tracking changes in trends of suicidality and is essential to ensure rapid responses as soon as incidence rates rise [3]. However, international comparisons of registrations have been challenging, and proper methods for registering non-suicidal self-harm and suicide attempts are still lacking [4].

In Denmark, suicide attempts have been registered and systematically validated since 1989 [5] to match the definition from WHO^1^ [6, 7]. These long-term register data are unique and invaluable as they contain detailed information that can be combined with data from the Danish National Patient Registry (DNPR) for use in suicide prevention [5]. The data validation process has revealed that around 30% of registered suicide attempts should be re-classified as habitual self-harming behaviour [8]. Another Danish study found that approximately 33% of attempted suicides were not identifiable as they were coded as accidents or similar [9]. This makes it impossible to retrieve reliable data from the suicide attempt codes alone. The same study found that the percentage of registered suicide attempts varied greatly within Denmark, but no further documented improvements have been made since then. Thus, far more than the estimated 6000 events registered with self-harm and suicide attempts in 2022 [10] could have occurred but are left unreported or incorrectly registered.

The DNPR classification systems for self-harm and suicide attempt have differed over time [11]. ICD-8 code E95 ‘suicide and self-inflicted injury’ [12] was used from 1977 to 1993 in combination with the Danish Classifications of Accidents in acute emergency departments, which since 1984 included the NOMESCO Classification of External Causes of Injury (NCECI) [11]. Intentional self-harm events are here coded under Reason for Contact (value = 4) [13]. Since 1994, the ICD-10 codes X60-84 ‘intentional self-harm’ have been used [14], primarily for psychiatry. The latest change occurred in 2019, when DNPR was updated to include separate codes for self-harm (ALCC05) and suicide attempt (ALCC04) in acute emergency departments [15, 16]. These classification changes complicate studies concerning registration practice.

The terms ‘suicide attempt’ and ‘non-suicidal self-injury’^2^ [17] have not yet been successfully separated in healthcare systems, so the extent of these problems continues to be reported incorrectly. This is important as people with habitual self-harm have different psychopathology to those who attempt suicide and may need different treatment approaches [4, 18].

The current study attempts to improve the quality and reliability of data registration across countries by examining how we can ensure the most valid data for suicide attempts in the Danish healthcare system. The study is expected to generate new information and recommendations for improving data collection practices in both Denmark and other countries. Based on semi-structured interviews with healthcare professionals from psychiatric and somatic emergency departments in Denmark, we investigated (i) the health professionals’ perceptions of registration practices for self-harm and suicide attempts and (ii) their suggestions for ensuring high-quality data. The study was limited to emergency departments as this is where patients with self-harm or suicide attempts are typically first seen.

## Methods

This qualitative study was reported using an adapted version of the Standards for Reporting Qualitative Research (SRQR) [19]. Semi-structured interviews were chosen to gain an in-depth understanding of the healthcare professionals’ perceptions. A conventional content analysis was chosen as existing theories on the topic were limited [20, 21].

### Setting

**Fig. 1:**
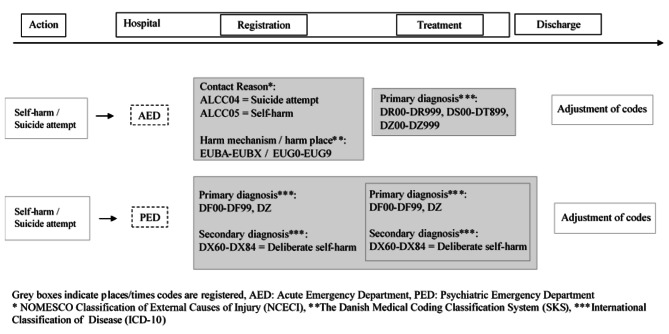
Procedures for registration of suicidal behaviour in emergency departments according to official guidelines

Acute emergency departments (AEDs) and psychiatric emergency departments (PEDs) frequently receive patients with either self-harm or suicide attempts. Registration practices differ in the Danish AEDs and PEDs, as detailed in Additional file 1 and summarized in Fig. 1. Furthermore, two different electronic health record systems are used in the regions of Denmark—the Health Platform (‘Sundhedsplatformen’) and the Electronic Patient Journal (‘Elektronisk Patientjournal/EPJ’). These two systems provide different registration options.

The Danish Health Data Authority is responsible for managing the DNPR and other administrative registries, while the Danish Clinical Quality Program-National Clinical Registries (RKKP) [22] manages registries that focus on disease-specific and procedure-specific quality [23]. RKKP began monitoring the suicide risk in patients with schizophrenia in 2022 [24].

The Danish healthcare system is free for all citizens and is financed from various tax-funded sources. To promote productivity and identical treatment across the Danish regions and across patient groups, activity-based subsidies were introduced as diagnosis-related groups, DRGs [25–27]. The DRG grouping can in principle influence registration practices, but it is no longer applied at hospital department level, being now only used in the economic flow from national to regional level [28, 29].

### Study participants

Healthcare professionals from AEDs and PEDs with any experience in registration practice were eligible for the study. This included medical secretaries, senior medical doctors (consultants), nurses, and registration advisers. The specific functions of the participants are described in Additional file 1 and are henceforth referred to as informants.

### Data collection

#### Sampling strategy

**Fig. 2:**
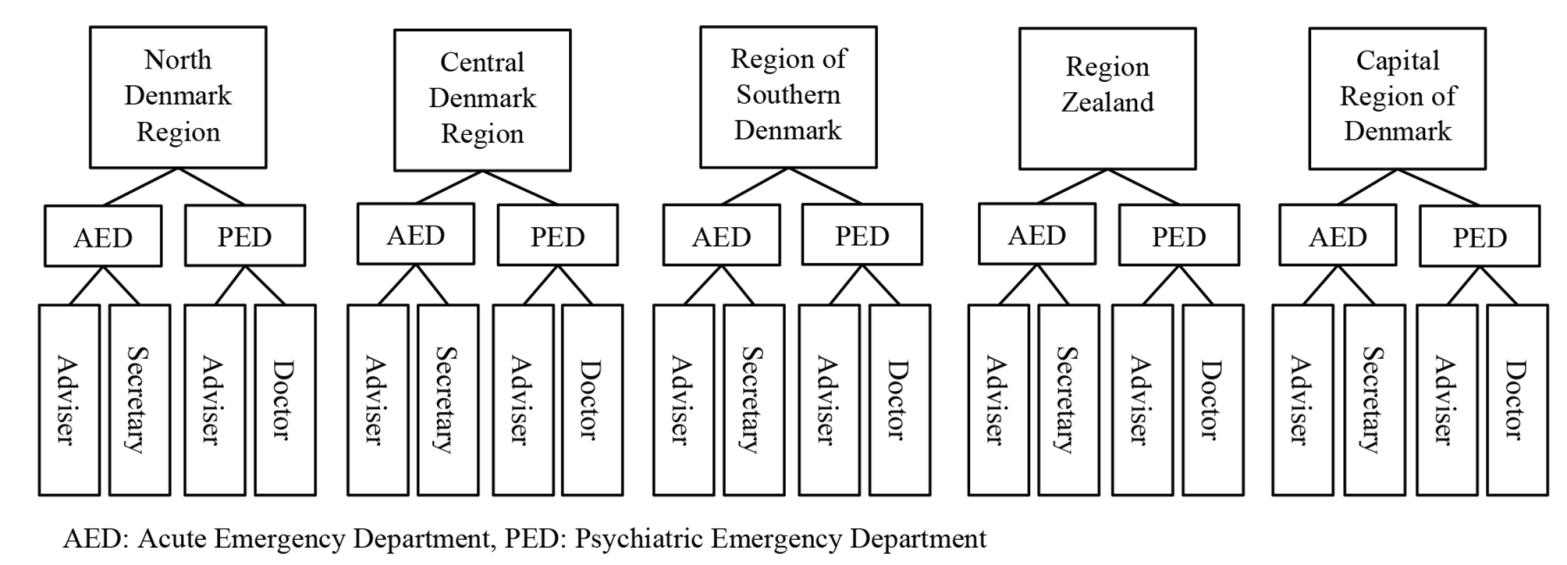
Sampling strategy of 20 expected informants

Interviews were planned in all five regions of Denmark to cover the variations in registration across the country. Snowball sampling was used to identify people who were knowledgeable about registration practice. The initial search strategy thus comprised contacting one AED and one PED (receiving patients > 18 years of age) in each of the five regions. A secretary was contacted in each AED, while a medical doctor or secretary was contacted in each PED depending on availability. Additionally, ten registration advisors were contacted—one specializing in registration in AED and one specializing in PED in each region. This approach resulted in 20 expected informants. A flowchart of the full sampling strategy is provided in Fig. 2.

Informants were localized through hospital websites, and initial contacts were asked to identify other healthcare professionals who knew about the registration codes. At a few of the EDs, nurses were also approached directly, resulting in two further interviews.

As the informants had busy work schedules, the interviews were mainly conducted via telephone or online meetings although the option to meet at the workplace was also offered. In two cases, two informants were interviewed together at their own request, and one ‘interview’ was conducted through e-mail correspondence. The sampling strategy was not completely successful in achieving full coverage from all five regions, but at least three different regions or types of healthcare professional were represented in each of the four categories (medical secretary or doctor + registration adviser in AED + PED). We considered this to be adequate to achieve data saturation after 49 contacts and 18 performed interviews.

### Data collection instruments and technologies

A semi-structured interview guide was developed with open-ended questions to ensure topics were fully covered and to enable conversations to develop. The interview guide was inspired by introductory meetings in the healthcare facilities and by the information contained in the registration instructions from the Health Data Authority [15]. All co-authors gave feedback on the draft interview guide.

The themes in the interview guide focused on (1) job tasks, (2) registration method, (3) quality assurance, (4) guidelines, (5) collaboration with other emergency departments, and (6) challenges and potential improvements in the registration process. The questions included in the interview guide are shown in Additional file 2. The interview guide was pilot-tested in two healthcare professional groups, one each from AED and PED. Due to the high quality of these interviews and no modifications being required, the data from these ‘pilot’ interviews were included in the study analysis.

The interviews were conducted between September 2022 and March 2023 and lasted between 27 and 63 min (average 38 min). The interviewer used a notebook as a supplement to record immediate reflections during or after each interview. The interviews were recorded using an audio recorder.

### Data processing and analysis

The interviews were fully transcribed using Word Dictation and were rewritten for accuracy by the first author. Data were anonymized immediately in all transcribed texts if names or places were mentioned.

The data were explored using content analysis, which is a systematic approach in which manifest and latent themes are identified through text condensation and interpretation [20, 30]. After the interviews had been read through, the following four steps were undertaken: (i) meaning units were identified and coded by the first author using NVivo 12, (ii) these meaning units were condensed according to their manifest content (the words used), (iii) the codes for the meaning units were compared and organized into categories, and (iv) themes were described based on an interpretation of the categories and the latent content (underlying meaning of the text).

After these steps, the second author read two raw interviews and took notes of identified themes. The first and second authors then discussed the themes and exchanged perspectives to check that they agreed on the themes to be taken forward. All quotes were shown to all co-authors to ensure agreement on the resulting themes.

## Results

A total of 20 informants were included in the study. As shown in Table 1, most of the informants were women, and the majority worked at psychiatric emergency departments.

**Table 1:** Characteristics of 20 included informants

Characteristics	Description (numbers)
Gender	Female (16) Male (4)
Age	28–63 years old, mean:47
Field	Psychiatric department (13) Somatic department (7)
Job title	Medical secretary (4) Consultant (6) Nurse (2) Registration adviser (8)
Experience in clinic	0.5–20 years, mean: 7.5

We identified six themes from the data collected in the interviews: (1) Variations in coding focus, (2) Coding inconsistencies across departments and staff, (3) Difficulties in determining the correct code, (4) Training and standardization of codes, (5) Guidelines and other suggested improvements, and (6) Collaboration challenges.

### Variations in coding focus

Data registration was generally perceived to be trustworthy in its basic form, such as having the correct patient ID attached to healthcare contacts. However, one registration adviser noted that uncertainty of information and classification errors increase with the total volume of required registration *(adviser, R4)*. When individuals with self-harm or suicide attempt present at an emergency department, the registration process is different depending on the type of setting.

#### AEDs

In AEDs, all types of patients arrive at any hour of day or night and often need rapid treatment. The focus when registering patients is typically on the physical symptoms and comes from a perspective of serious diseases or accidents:

*‘I think I have been bad at using [codes for] self-harm and suicide attempts. Yeah, because that’s just the way, you know. I’m taught the other way [with accident coding]’ (secretary, R10)*.

*‘[The reliability of data for registered suicide attempt] is definitely not good. And it’s never been a focus area for us… in somatics… (…) they’re so busy, so I don’t think they say, “now we just focus on this…”’ (adviser, R9)*.

At the same time, all informants considered registration to be important for the purpose of statistics and scientific work as well as ‘*improving the patients and our working conditions’ (Secretary, R2).* The data are also perceived to be useful for prevention, but then sufficient resources are required or expected to fully utilize the data *(Consultant, R14)*.

#### PEDs

The focus in the PED is on mental disorders, and events with self-harm and suicidal behaviour are common scenarios in the everyday work. The staff are trained to help patients by assessing the mental disorder and the treatment options. This is reflected in their approach to registration, where they have the events as supplementary diagnoses:

*‘I also think part of it is that X-codes must not really be a primary diagnosis code – and often, that is the one we show what they are here for (…) Then the problem also becomes that we don’t really focus [on the self-harming event] - ‘well okay, it’s schizophrenia or it’s depression’ and then… yeah’ (Consultant, R13)*.

*‘Um, it won’t be done. No. So, of course we do suicide risk assessment (…), but we’re not very good at putting [secondary] diagnosis codes on (…) For us it is probably not so important whether it is registered completely correct, because we can always read the journal, and if it is someone who comes several times a month, we know the person’ (Consultant, R19)*.

The overall response from PED informants was that self-harm and suicide attempts are often left unregistered and these codes are not required in the system. A few informants mentioned the ICD-10 code Z032 (‘Observation for suspected mental and behavioural disorders’) as a substitute code used together with notes of the event, although this code was originally intended to be used only until a disorder is clarified *(Consultant, R14)*. Another reason for unregistered events could be a nurse conversing with a patient who had self-harmed but had decided to go home before journal registration had taken place *(Nurse, R1)*.

In both AEDs and PEDs, the overall tendency was that codes were often not used for events of self-harm and suicide attempt.

### Coding inconsistencies across departments and staff

Secretaries, consultants, nurses, and ambulance staff can all participate in the initial registration of an event. If insufficient details are given about suicidal behaviour, problems can arise later when secretaries check the registrations for data quality. This can cause errors and direct loss of information in the system.

#### AEDs

*‘Well, the error often happens because (…) the only thing I think [the nurses] have been told to register is just when it happened, i.e. the time of the accident… And everything else they leave blank’ (Secretary, R10)*.

*‘(…) [if a patient] comes in with the ambulance, then they are not registered in the emergency room as a suicide attempt, they are registered as an accident. And then it is only (), when we handle the injury report (…), that they are registered as a suicide attempt (…). But I actually think there is a bit of a miss there (…) [because] we have to [be aware and] manually change something that is already registered (…)’ (Secretary, R17)*.

This scenario where corrections should be made later on by other health care professionals is only attainable if the journals are ‘*fed with the right information’ (Adviser, R18)*.

Different registration cultures could be observed within the same hospital organization, where each department can have its own way of registering codes, e.g. for unknown reasons, one medical department was said to consistently record all contacts as disease *(Secretary, R10)*. The secretaries usually view the patient’s journal before it is closed, but new information about suicidal behaviour in the patient history will not necessarily change the registration data:

*‘We are very adamant that the arrival registration is based on the patient’s own story. Then they can give a different explanation to the doctor, and we do not then change our write-up, because that is what they have told us.’ (Secretary, R2)*.

#### PEDs

The registration culture in PEDs appeared to vary across departments and regions due to differing policies. Some regions have secretaries to register coding while other regions have delegated the job to medical doctors, although this switch does not seem to work out as well as expected. While most places double-check the data registrations, a few places did not have enough resources for this:

*‘No, [quality assurance] we don’t have that. We lack specialists in psychiatry to a great extent. So we don’t have the resources for that.’ (Consultant, R19)*.

*‘I think what sort of happened before… it was that the doctor dictated… and… there was someone who always knew everything in the ward and that was the secretary because she sat and wrote all the notes, right, and since she wrote the notes, well, she could just put the code on, right?’ (Consultant, R13) ‘[To have a secretary] would actually be the best thing’ (Consultant, R19)*.

*‘There are so many [faulty discharge summaries for approval] that I have given up on informing the junior doctors unless it is completely out of the blue and could have consequences. It is simply too time-consuming and should be something that came from a central team, i.e. introduction, training, persistence from the functional managers in the sections or departments where the individual doctor is employed.’ (Consultant, R20)*.

Even when the data are double-checked, ‘*data are no better than what is being recorded*’ *(Adviser, R3)*, meaning that if the information about a suicide attempt is insufficiently registered, codes will not be added later on. Only one informant mentioned a solution to this issue:

*‘There is a [patient] course description called “suicidality or self-harm” [AAF18C] and (…) if the patient is in this process description, then they must also have a second diagnosis that states that there has been some self-harm. And then the departments have the opportunity to go in and check and say ‘oops, we actually need to make a [secondary] diagnosis’, right?’ (Adviser, R4).*

### Difficulties in determining the correct code

Although self-harm and suicide attempt are not always registered according to the official procedure, most of the informants had considered the importance of codes and the registration impact:

#### AEDs

*‘So, we ask – that is if it is appropriate for the patient – if it was intended that they would commit suicide and if not then… in this way we separate the two codes’ (Secretary, R2)*.

*‘I would find it very difficult to write [it as] a suicide attempt if I am not absolutely 100% sure that this is what happened’ (Secretary, R10) ‘… after all, you don’t want to put a stamp on a person about suicide or self-harm if it’s not true’ (Secretary, R12)*.

*‘The one I’ve corresponded with (…), she was very surprised that aspirin [overdose] cases were part of self-harm [coding], so they just hadn’t used it at all… I could imagine’ (Adviser, R9)*.

The staff in the AEDs wished to avoid labelling someone incorrectly, and even if they suspected self-harm, they were more likely to code it as an accident if the patient phrased it as such. They found it sometimes challenging to ask a vulnerable patient about their cause of hospital contact and so might use a more neutral registration category such as accident to avoid asking uncomfortable questions about the real cause.

#### PEDs

The approach in the PED was usually different as the staff learn more details about the patient through the mandatory suicide risk assessment. It could still be difficult to separate patient behaviours into different codes, however.

*‘There are an enormous number of codes and so it is, among other things, one of the reasons that in everyday life, that it is not certain that it will be exactly as you wanted it to be… Because it is EXTREMELY comprehensive [and difficult] to familiarize yourself with: Is it exactly a X6020 or 21 or is it actually over in a 6120. It’s not easy. (…) then I imagine that you panic and choose one or the other. It sounds terrible, but maybe you drop it altogether because it’s too difficult’ (adviser, R4)*.

*‘There are some obvious problems with how to code (…). If a person with severe personality disorder says that [their intention] was to die, but he/she has done it 20 times before, then one must assume that he/she knows very well that it does not lead to death (…). What if a “goodbye” message was sent at the same time (…)? I know from discussions with others that there are different views on it.’ (Consultant, R20)*.

The more experienced the healthcare professional is with the patient group, the more they appear to become accustomed to overruling the patient´s words if the professional assesses the situation differently. This could be a habitual self-harming event with a nearly fatal outcome that could be judged too serious to code as self-harm only, despite the patient saying otherwise. The registration procedure relies on subjective interpretations of the patient’s behaviour, and it seems that better ways to operationalize the terms and codes are desired *(Consultant, R13)*. Some healthcare professionals will only code an event if physical marks exist while others will code it regardless. The long list of code combinations complicates the registration when the healthcare focus is on the patient and other patient management tasks. This lack of focus can also result in juxtaposition errors, e.g. if the adjacent code is marked in error.

### Training and standardization of codes

There appeared to be a clear difference between the secretaries and the other staff in relation to coding practice. The secretaries have been trained in coding, but the doctors and nurses concentrate on patient care and treatment. It seemed that the secretaries were also more capable of handling the more complex registration codes.

#### AEDs

*‘(…) we would actually really like to be allowed to register them as [secondary] diagnosis [X60-84]. We have been able to do that in the past.’ (Secretary, R12)*.

*‘I think a [requirement] is missing in the system, when we have put the suicide code on, and we use the category of medical poisoning with medicine. (…) you [should] have to fill in the medicine.’ (Secretary, R17)*.

Several informants mentioned that doctors often use the X60-84 codes in AEDs although they are not allowed to, as these codes should only be used in psychiatry. Therefore, these codes end up on an error list. Allowing the same type of codes in both AEDs and PEDs would simplify the registration practice across the sectors and for all professional groups.

#### PEDs

A few informants in the PED mentioned that the DRG activity-based subsidies can sometimes influence registration practice. Similarly, the requirement to record data for the RKKP clinical quality registry can have a negative effect on registration.

*‘But in addition, we are not DRG-regulated in psychiatry either, so… I don’t really have that much focus on… getting the code totally correct’ (Consultant, R13)*.

*‘(…) you can say that all regions, they are competitive, they want to do well after all. And who is it that… like being punished for having a good record of suicide attempts? (…) So, why should we… invest in being punished?’ (Consultant, R12, about registration requirements to RKKP in relation to schizophrenia).*

### Guidelines and other suggested improvements

#### AEDs

The secretaries explained that when a new secretary starts working in a somatic department, they receive peer-to-peer training. Only a few hospitals appeared to provide specific training courses for secretaries. Therefore, they depend on the induction training from their peers but can still have questions about local practice in the department:

*‘When you have to teach new ones () often [they ask], so “what should I code here?”. I’m just as doubtful as they are.(…) Really, if you only had something [to refer to].’ (Secretary, R10)*.

*‘Self-harm falls under the upper group called “suicide”. (…) If [it was] changed to suicide/self-harm, I’m sure more [events] would be registered. Because I may have a suspicion that everything is not registered because when you select ‘suicide’… This is not what we typically have in an emergency department’ (Secretary, R12)*.

Informants suggested that the registration of suicidal behaviour could be improved, e.g. by having reminder post-its near the computer screens and providing more detailed coding manuals, supplemented by regular informative mails and meetings about expected registration practice. An improved setting for the initial communication between the patient and the staff was also mentioned, e.g. to provide more privacy to talk about the reason for the AED visit.

‘*It wouldn’t require much internally in our secretarial group for someone to stand up… to a staff meeting, or write it out in a newsletter’ (Secretary, R10)*.

*‘So, I’m thinking in terms of discretion and things like that, because maybe it’s a bit like, standing in a queue [in the AED] and then there are a lot of people and having to say “Yes, I’m cutting myself“ or “I just tried this and that”… In other words, not many people want to… think it was particularly cool… so it may well be… that… [questionnaire or similar] could do something, in the way that it could be more confidential to…‘ (Secretary, R10)*.

*‘So we just sit inside such a… bubble [of plastic shields]… In other words, we can hardly hear what is being said (…) and it just already creates some kind of strange distance there’ (Secretary, R10)*.

#### PEDs

Many of the PED informants did not know about the official guideline document for registrations, but some had gained knowledge from an affiliated registration adviser visiting their ward. Emails about registration practice regarding suicide behaviour helped in one instance to create a motivational change in the daily work procedure. Other suggestions were made for changing the procedure, such as screening or confirming a suicide attempt differently in the system:

*‘It’s just printed from some [long] list and then hung-up and… () Well, we don’t have the opportunity to bring that up every time we have a patient who comes in with a suicide attempt.’ (Consultant, R19)*.

*‘Otherwise, I don’t know how you would be reminded… you would have to do a completely different kind of registration, where you use procedure code instead… I don’t know if it would be any better… but then it’s not just the doctors, it’s all the staff who can go in and put these codes on.’ (Adviser, R5)*.

*‘The problem is that when we have added (…) a [secondary] diagnosis, then it will also be continued (… ) so it’s not just an under-registration, sometimes it can be an over-registration because they’ve kind of put it on and then, uh… next time, they won’t… fix it, [by] pressing release, to discontinue the code’ (Consultant, R13)*.

### Collaboration challenges

The electronic patient journal can be accessed by all hospital departments in all regions, hence the interest in collaboration between the AEDs and PEDs. Some informants mentioned joint meetings and the possibility of sending staff to the other emergency department when needed if the departments are close enough in proximity. Informants working in emergency departments that are far from their AED or PED counterpart often mentioned a lack of connection and collaboration. Most of the informants had considerable working experience in their field, but a few informants made assumptions about the registration practice without deeper knowledge on the topic. One informant was uncertain how the statistics are calculated when a patient with suicide behaviour is registered at both an AED and a PED:

#### AEDs

*‘*W*e do not get any messages back, now let’s say that we have misregistered someone for an accident and then they go to the PED, and they discover that it is a suicide attempt or similar. Then we do not get any feedback about changing it’ (Secretary, R17)*.

#### PEDs

*‘We do not have the opportunity to correct their [the AED’s] diagnoses, unfortunately (…) but the problem is when they start messing around with our diagnoses [e.g. on personality disorders], which they shouldn’t do, it usually always goes wrong.’ (Consultant, R14)*.

*‘If they go to the AED, and get some treatment there, and then come to us, () if we then also code it, is it then… has there been two incidents, or was there really only one incident?’ (Consultant, R13)*.

Informants from both the AED and PED expressed uncertainty about the collaboration on data, indicating a need to improve communication when linking patients’ journals across the somatic and psychiatric sectors.

## Discussion

We believe this to be the first qualitative study to investigate healthcare professionals’ perceptions of registration practice for self-harm and suicide attempts. Our study contributes valuable information about the unsystematic and insufficient registration of self-harm and suicide attempts in hospital emergency departments. This is likely to result in unreliable healthcare data on these events. Our interview informants suggested several ways in which registration practice could be improved, such as by making registration codes more visible in documents near the computer, increasing the technical assistance provided in the software, and ensuring sufficient medical secretary staff to manage the coding system.

There are several issues to highlight from this study. First, it is important to ensure valid and reliable data in the national patient registers as these data are used in epidemiological studies and national health statistics to investigate health determinants that play a part in health policies and prevention strategies. Because the Danish patient data are extremely comprehensive, they are widely used and generally accepted as being of high quality. However, the completeness and validity of patient registers appear to vary [31], and even more attention to detail has been recommended if these data are to be used for registry research [11, 32]. Underreporting in data on self-harm and suicide attempts has been identified previously [9, 33], but earlier recommendations for improvements such as clearer instructions and common codes in AEDs and PEDs [9] have not been adequately implemented. The formal separation of ‘intentional suicide attempts’ and ‘habitual self-harm’ was introduced to AEDs in Denmark in 2019, and a preliminary analysis from Odense University Hospital showed that misclassification of ‘injury’ and ‘intentional suicide attempts’ has diminished substantially since then [34]. However, there is still a great need for more visible instructions and clearer procedures, especially for medical secretaries. It would seem that such relatively small efforts could result in much more valid and useful data.

Medical secretaries already play an important role in ensuring the quality of registrations due to their situational knowledge from clinical, administrative, and organizational insights, which the doctors do not have to the same degree [35]. Secretaries are highly focused on coding practices, but limitations can still arise in the communication with patients, for example if the patient omits the truth to avoid stigmatization [36]. It is possible that electronic patient self-registration (as opposed to via the secretary) could help to improve data accuracy [37] as well as department productivity [38]. This sort of development would seem achievable given the Danish digital health strategy and the objective of patient-centred care [39]. A review of the nurses’ role could also lead to improved coding and registration practices in both AEDs and PEDs, alongside a sufficient number of medical secretaries [35]. Based on the information from our nurse informants, nurses appear to have both the ability and the desire to help more with the coding, and this could release resources from other staff and increase the chance of correct data registration.

Secondly, the definitions for suicide attempt and self-harm seem complicated to apply in clinical practice. The organization’s culture and the staffs’ subjective assessments play a significant role in the judgement of an event. Should it be phrased as a suicide attempt if a patient stood on a bridge for a long time, or are body marks perceived as a prerequisite? If habitual self-harming behaviour has been observed, but a suicide note is found, is it then an attempt or a way to get attention? And should lethal methods of self-harm be perceived as accidents? In these cases, more guideline examples could be helpful for healthcare staff. WHO has already published a practice manual for maintenance of surveillance systems for suicide attempts and self-harm [2], but further training is advisable. The relevant ICD-10 codes used in PEDs contain 25 different method categories for suicide behaviour (X60-84) that again should be combined with a digit to reveal if it is self-harm, suicide attempt, or suicide and another digit to indicate whether it occurred before or after admission. This results in 125 unique codes, which can seem overcomplicated compared to using separate binary codes.

Finally, more knowledge about the collaboration and data flow across departments would be useful. Several informants expressed little knowledge or communication about data generated between the AED and the PED. Occasional short meetings could limit the frustrations from incorrect coding that cannot be changed elsewhere and might improve the feedback and collaboration between the departments involved. A ‘social track’ has been suggested [40] to strengthen patient care in suicidal behaviour across the AED and the PED. This could be a way to promote the needed teamwork across sectors.

While an activity-based subsidy (DRG) has been used to increase activities in the Danish healthcare sector [29, 41], disadvantages become evident in areas that were not included in the agreement. It would be interesting to investigate whether the coding of self-harm and suicide attempt increases in the EDs if the codes for self-harm and suicide attempts were included in a DRG-regulation or similar. However, strict DRG-based budgets have not been applied at the department level since 2019 [26].

At the patient level, the improvements in registration practice could result in more systematic and specialized treatment as well as prevention options. If self-harm and suicide attempt are more easily identified from each other and from other similar codes such as accidents, more valid information would be available for studying patient profiles and individual needs in treatment.

### Methodological considerations

The included informants only represented one type of healthcare professional per region in each somatic and psychiatric department, making it impossible to generalize to all regional departments. However, the same overall findings were found in all participating departments. Other healthcare professionals such as doctors from the AED or pre-hospital ambulance staff could also have been interesting to include in the study.

The interviewer had limited insights into clinical practice, making it difficult to gain full understanding of the organizational structures. An advantage of this was that informants would explain things in more depth rather than perceiving certain conversation subjects as implicitly understood.

Telephone and online meetings can complicate communication, especially when it is impossible to read the body language or if both interviewer and informant speak at the same time making the recording quality poorer. On the other hand, these flexible approaches increase the likelihood of informants agreeing to be interviewed during a busy workday.

## Conclusions

This study provides essential new knowledge about registration of suicide attempts and self-harm in emergency departments. Although efforts have been made to improve registration practice in emergency departments following the recommendations from Danish studies since 2006 and from WHO in 2016, it appears that more improvements are needed.

The healthcare professionals interviewed in this study did not perceive the registered data on self-harm and suicide attempts to be reliable, and they had several suggestions for how to improve registration practice, such as better tools to ensure correct coding. The availability of more precise data for research purposes would lead to greater opportunities to provide patients with relevant specialized treatment and would assist in planning suicide prevention strategies.

### Implications for clinical practice

The study findings have resulted in a number of concrete suggestions for future registration practice in AEDs and PEDs of self-harm events and suicide attempts. It appears that the validity and reliability of such data could be improved by:

- Ensuring visible and simple instructions for all relevant healthcare professionals, including definitions and examples of how to distinguish between self-harm and suicide attempt. Training courses and systematic induction training for new employees could help to standardize peer-to-peer training.
- The opportunity for all EDs to employ a medical secretary or similar to perform quality assurance on coding data.
- Disseminating useful coding information so that the staff can double-check that relevant diagnoses are applied, such as ‘AAF18C’ in PEDs.
- Testing alternatives for patient check-in options that include more anonymous recording of the contact reason and removing redundant screens between secretaries and patients to ease communication.
- Random checks on how contacts are registered, based on a list of variables for evaluation that could highlight coding relevancy.
- Improving data flow between the AED and PED by having structured collaboration at both individual patient level and overall organizational level.

*(For higher political levels)*

- Discussion of code refinements that could be used across sectors by more healthcare professionals; to avoid over-reporting of the same event, codes should automatically discontinue for each new contact.
- Consideration of financial incentives for correct coding of self-harm events and suicide attempts.

### Electronic supplementary material

Below is the link to the electronic supplementary material.

**Supplementary Material 1.** Additional file (1) Context.Description of AEDs / PEDs and job tasks of the health care professionals.

**Supplementary Material 2.** Additional file (2) Interview guide. Description of themes and questions used in the data collection.

## Data Availability

Restrictions apply to the availability of the study data as they were used under license for the current study and so are not publicly available. However, data are available from the authors upon reasonable request and with permission of OPEN.

## Footnotes

1 ‘a*n act with a non-fatal outcome, in which an individual deliberately initiates a non-habitual behavior that without intervention from others will cause self-harm, or deliberately ingests a substance in excess of the prescribed or generally recognized therapeutic dosage, and which is aimed at realizing changes which the subject desired via the actual or expected physical consequences’*.

2 *‘the direct, deliberate destruction of one’s own body tissue in the absence of intent to die’*.

## Declarations

### Authors’ information

The first author, a PhD student and researcher in public health, conducted all the interviews. The other members of the research team were an epidemiologist, a sociologist, a psychiatric consultant, and a somatic consultant. The first and last authors participated in an introductory meeting with healthcare staff about the organization of healthcare in somatic and psychiatric facilities in one region before the interviews were performed.

## Abbreviations

AED: Acute emergency department
DNPR: Danish National Patient Register
DRG: Diagnosis-related groups
ED: Emergency department
ICD: International Classification of Diseases
PED: Psychiatric emergency department
RKKP: Danish Clinical Quality Program-National Clinical Registries
WHO: World Health Organization
