# Supplementary material for: Opening the black box of registration practice for self-harm and suicide attempts in emergency departments: a qualitative study
Source: BMC Prim Care. 2024 Apr 27;25:139. doi: 10.1186/s12875-024-02393-6 (PMC11055235; doi:10.1186/s12875-024-02393-6)
Supplement: Supplementary file 1 — Supplementary Material 1. Additional file (1) Context.Description of AEDs / PEDs and job tasks of the health care professionals. [file 12875_2024_2393_MOESM1_ESM.docx]

# **Interview guide**

**[Introduction text about the project]**

**Demographic data:**

Geographic area:

Job title:

Number of month/years as [job title] / in clinic:

Age and sex:

The first questions concern the place and type of patients:

1. **What does your work in the department consist of?**

*Prompts:*

*• What is the procedure in relation to patients with suicidal behavior?*

*• How to define self-harm versus suicide attempt?*

*• How often do you experience patients with suicidal behavior?*

*• Any dilemmas related to management of these patients?*

The next questions concern registration of self-harm/suicide attempts.

1. **In your opinion, what is the purpose of registration?**

*Prompt:*

*• How reliable are data?*

1. **What happens when a patient presents with self-harm?**

*Prompts:*

*• Are codes or text registered? If codes, how are they located?*

*• Is the method (and time of event) registered in any way?*

*• What happens in case of uncertainty about the event?*

1. **Is there any quality assurance?**

*Prompts:*

*• Can codes be changed later on?*

*• Do other people double check the codes?*

1. **Is there guidance on how the registration practice should be?**

*Prompts:*

*• Are there any training / courses / education / manuals available?*

*• Have there been any changes in the codes or system during time?*

1. **How is the cooperation with the nearest (Acute or Psychiatric) Emergency Department regarding these patients?**

*Prompt:*

*• Can data come from ambulances or other units?*

1. **Do you see any challenges with the current registration practice?**

*Prompt:*

*• What are your suggestions for improvement?*

1. **Do you have anything else to add regarding what we have been around - or need to get around?**
