# Supplementary material for: Opening the black box of registration practice for self-harm and suicide attempts in emergency departments: a qualitative study
Source: BMC Prim Care. 2024 Apr 27;25:139. doi: 10.1186/s12875-024-02393-6 (PMC11055235; doi:10.1186/s12875-024-02393-6)
Supplement: Supplementary file 2 — Supplementary Material 2. Additional file (2) Interview guide. Description of themes and questions used in the data collection. [file 12875_2024_2393_MOESM2_ESM.docx]

**Context**

#### AEDs

When a new patient presents to the AED, a receptionist (medical secretary or nurse) will initially register a Reason for Contact that includes nine different codes in the NOMESCO Classification of External Causes of Injury (NCECI). This mainly covers disease, injury, violence, self-harm (since 2019), and suicide attempt (1).

In the reporting instructions from the Danish Health Data Agency, a suicide attempt is defined in accordance with the WHO definition, while self-harm is described as a deliberately intended action that does not meet the suicide attempt criteria (1). Examples of self-harm are given as cutting or poisoning with painkillers. When registration of self-harm or suicide attempt occurs, it is possible to record the harm mechanism and location of event using the Danish Medical Coding Classification System (SKS-codes). Examples of these codes are EUBA: ‘Fall and jump in level or from under 1 meter’ and EUG0: ‘Public road and parking area’. It is also possible to specify any involved product in the harm mechanism such as EUYR6005 for ‘paracetamol’. The time of both entry and action is also registered, and if the time of action is unknown, entry time should be stated twice (1, 2).

A medical doctor will see the patient and give a Primary Diagnosis from the International Classification of Diseases (ICD) to describe the sort of injury sustained, for example DT39 ‘Poisoning by nonopioid analgesics, antipyretics and antirheumatics’ or DS61 ‘Open wound of wrist and hand’ (3). If any new information is revealed about the action, i.e. being a suicide attempt and not an injury, there is an instruction to change the initial coding. The patient will either be discharged or directed to a PED for further assistance.

#### PEDs

A receptionist (medical secretary or nurse) will evaluate the patient’s needs, and a nurse will make a suicide risk assessment. According to the official guideline for registration, the procedure is to register a Primary Diagnosis from ICD, chapter 5 (F codes) or chapter 21 (Z codes) that relate to either ‘Mental, behavioural, and neurodevelopmental disorders’ or ‘Factors influencing health status and contact with health services’ (1, 3). A sub-diagnosis can be added, which in case of self-harm or suicide attempt will be the ICD-10 codes X60-84 indicating ‘Intentional self-harm’. This coding contains extra digits, differentiating between self-harm, suicide attempt, and suicide, but also information about whether the action occurred before or after entry (e.g. X78*21* being ‘*Self-harm* by sharp object *during* admission’ and X78*10* being ‘*Suicide attempt* by sharp object *before* admission’).

**Study participants**

#### Medical secretary

The medical secretary usually receives patients in the reception and registers all the necessary information and introductory codes in the journal system. The secretary also answers phone calls from patients and writes out the journal notes from the doctor’s dictation of the patient’s treatment and procedures. The medical secretary is also responsible for correcting any errors generated by the system whenever invalid codes have been used.

#### Nurse

The nurses usually focus on the patient’s wellbeing and are not assigned to the same registration requirements as medical secretaries or medical doctors.

#### Medical doctor

Medical doctors examine and treat patients and then either dictate their notes to secretaries or describe the patient encounter directly in the electronic system without further assistance. Consultants can have additional tasks such as supervision of junior doctors or managerial responsibilities such as approving discharge summaries.

#### Registration adviser

The registration advisers (also referred to as advisers) help staff to improve the quality of registrations. They visit the hospital departments and provide further training to staff when necessary. They also look at trends in registered data and ensure that departments have correct and updated information from the Danish Health Data Agency.

**References**

1. Sundhedsdatastyrelsen. Indberetningsvejledning til Landspatientregistere. 2023.

2. SKS-browser, vers 4.06 [Internet]. 2023. Available from: <https://medinfo.dk/sks/brows.php>.

3. World Health Organization. International Statistical Classification of Diseases and Related Health Problems 10th Revision (ICD-10) 2019 [Available from: <https://icd.who.int/browse10/2019/en>.
